# Brain Tumor Classification from MRI Using Image Enhancement and Convolutional Neural Network Techniques

**DOI:** 10.3390/brainsci13091320

**Published:** 2023-09-14

**Authors:** Zahid Rasheed, Yong-Kui Ma, Inam Ullah, Yazeed Yasin Ghadi, Muhammad Zubair Khan, Muhammad Abbas Khan, Akmalbek Abdusalomov, Fayez Alqahtani, Ahmed M. Shehata

**Affiliations:** 1 School of Electronics and Information Engineering, Harbin Institute of Technology, Harbin 150001, China; 2 Department of Computer Engineering, Gachon University, Sujeong-gu, Seongnam-si 13120, Republic of Korea; 3 Department of Computer Science, Al Ain University, Abu Dhabi P.O. Box 112612, United Arab Emirates; 4 Faculty of Basic Sciences, Balochistan University of Information Technology Engineering and Management Sciences, Quetta 87300, Pakistan; 5 Department of Electrical Engineering, Balochistan University of Information Technology, Engineering and Management Sciences, Quetta 87300, Pakistan; 6 Department of Artificial Intelligence, Tashkent State University of Economics, Tashkent 100066, Uzbekistan; bobomirzaevich@gmail.com; 7 Software Engineering Department, College of Computer and Information Sciences, King Saud University, Riyadh 12372, Saudi Arabia; 8 Computer Science and Engineering Department, Faculty of Electronic Engineering, Menoufia University, Menofia 32511, Egypt

**Keywords:** deep learning, brain tumor, magnetic resonance imaging, classification, neural network, pre-trained models, healthcare

## Abstract

The independent detection and classification of brain malignancies using magnetic resonance imaging (MRI) can present challenges and the potential for error due to the intricate nature and time-consuming process involved. The complexity of the brain tumor identification process primarily stems from the need for a comprehensive evaluation spanning multiple modules. The advancement of deep learning (DL) has facilitated the emergence of automated medical image processing and diagnostics solutions, thereby offering a potential resolution to this issue. Convolutional neural networks (CNNs) represent a prominent methodology in visual learning and image categorization. The present study introduces a novel methodology integrating image enhancement techniques, specifically, Gaussian-blur-based sharpening and Adaptive Histogram Equalization using CLAHE, with the proposed model. This approach aims to effectively classify different categories of brain tumors, including glioma, meningioma, and pituitary tumor, as well as cases without tumors. The algorithm underwent comprehensive testing using benchmarked data from the published literature, and the results were compared with pre-trained models, including VGG16, ResNet50, VGG19, InceptionV3, and MobileNetV2. The experimental findings of the proposed method demonstrated a noteworthy classification accuracy of 97.84%, a precision success rate of 97.85%, a recall rate of 97.85%, and an F1-score of 97.90%. The results presented in this study showcase the exceptional accuracy of the proposed methodology in accurately classifying the most commonly occurring brain tumor types. The technique exhibited commendable generalization properties, rendering it a valuable asset in medicine for aiding physicians in making precise and proficient brain diagnoses.

## 1. Introduction

The development of a brain tumor can occur when there is an abnormal proliferation of cells within the brain tissues. Tumors have been identified by the World Health Organization (WHO) as the second most significant contributor to global mortality [1,2]. Brain tumors can be categorized into two main types: benign and malignant. In most instances, benign tumors are not considered a substantial risk to an individual’s health. It is primarily due to their comparatively slower growth rate than malignant tumors, lack of ability to infiltrate adjacent tissues or cells, and inability to metastasize. Their recurrence is generally uncommon after the surgical removal of benign tumors.

Compared to benign tumors, malignant tumors can infiltrate adjacent tissues and organs, and if not promptly and effectively managed, they can result in significant physiological dysfunction. Detecting brain tumors in their earliest stages is crucial for optimizing the survival rate of patients. Gliomas, meningioma, and pituitary tumors are the three most frequently diagnosed types of brain tumors. Glioma is a neoplasm originating from the glial cells that encompass and provide support to neurons. The cellular composition of these structures includes astrocytes, oligodendrocytes, and ependymal cells. A pituitary tumor is formed within the pituitary gland. A meningioma is a tumor originating within the meninges, the three layers of tissue between the skull and the brain. According to the cited source, it has been established that meningiomas are classified as benign tumors, while gliomas are categorized as malignant tumors. Additionally, pituitary tumors have been identified as benign. The dissimilarity above represents the most notable differentiation among these three cancer variants [3,4,5].

Various symptoms can be produced by benign and malignant brain tumors, depending on factors such as their size, location, and growth rate. The symptoms of primary brain tumors may exhibit variability among individual patients. Glioma has the potential to induce various symptoms, including aphasia, visual impairments or loss, cognitive impairments, difficulties with walking or balance, and other associated manifestations. A meningioma is often associated with mild symptoms, including visual disturbances and morning migraines. Pituitary tumors can exert pressure on the optic nerve, leading to symptoms such as migraines, vision disorders, and diplopia [6,7].

Hence, it is imperative to distinguish among these diverse tumor classifications to precisely diagnose a patient and determine the optimal course of treatment. The expertise of radiologists significantly influences the speed at which they can detect brain malignancies. Although magnetic resonance imaging (MRI) presents challenges due to its dependence on human interpretation and the complexity of processing large volumes of data, it is commonly employed to categorize different forms of cancer. Biopsies are commonly employed in identifying and managing brain lesions, although their utilization before definitive brain surgery is infrequent. Developing a comprehensive diagnostic instrument for detecting and classifying tumors based on MR images is imperative [8]. The implementation of this approach will effectively mitigate the occurrence of excessive operations and uphold the impartiality of the diagnostic procedure. The healthcare industry has been significantly influenced by recent technological advancements, particularly in the fields of artificial intelligence (AI) and machine learning (ML) [9,10,11,12]. Solutions to various healthcare challenges, such as imaging, have been successfully identified [13,14,15,16,17,18]. Various machine-learning techniques have been developed to provide radiologists with unusual insights into the recognition and classification of MR images. Medical imaging techniques are widely recognized as highly effective and widely utilized modalities for cancer detection. These methodologies facilitate the identification and detection of malignant neoplasms. The methodology holds significance due to its non-invasive nature, as it does not require invasive procedures [19,20].

MRI and other imaging modalities are commonly employed in medical interventions because they produce distinct visual representations of brain tissue, facilitating the identification and categorization of diverse brain malignancies. Brain tumors exhibit various sizes, dimensions, and densities [21]. Moreover, it is worth noting that tumors can exhibit similar appearances, even when they possess distinct pathogenic characteristics. A substantial quantity of images within the database posed a significant challenge in classifying MR images utilizing specialized neural networks. Due to the ability to generate MR images in multiple planes, there is a potential for increased database sizes. In order to obtain the desired classification outcome, it is necessary to preprocess MR images before integrating them into different networks. The Convolutional Neural Network (CNN) is employed to solve this problem, benefiting from several advantages, such as reduced preprocessing and feature engineering requirements. A network with lower complexity necessitates a reduced allocation of resources for implementation and training compared to one with higher complexity. Resource limitations hinder the utilization of the system for medical diagnostics or on mobile platforms. The method must be relevant to brain disorders for daily regular clinical diagnosis.

The main contributions to this investigation are delineated as follows:

- This study presents a novel methodology integrating Gaussian-blur-based sharpening and Contrast-Limited Adaptive Histogram Equalization (CLAHE) with the proposed model to facilitate more precise diagnostic procedures for identifying glioma, meningioma, pituitary tumors, and cases without malignancies.
- This investigation aims to demonstrate the superiority of the proposed methodology above existing methodologies while highlighting its ability to achieve comparable results with fewer resources. Additionally, an assessment is conducted on the network’s potential for integration into clinical research endeavors.
- The results obtained from this analysis demonstrate that the novel strategy surpasses previous methodologies, as indicated by its ability to attain the highest levels of accuracy on benchmark datasets. Further, we evaluate the prediction capabilities of this strategy by comparing it to pre-trained models and other established strategies.

The subsequent sections of this work delineate the literature review in Section 2. Section 3 explores the dataset, methodology, optimization techniques, and pre-trained models. Section 4 presents the findings obtained from the conducted experiments. Section 5 involves a discussion. Lastly, Section 6 provides a conclusive summary.

## 2. Literature Review

It is challenging to distinguish between various varieties of brain tumors. The authors [22] examined the clinical applications of DL in radiography and outlined the processes necessary for a DL project in this discipline. They also discussed the potential clinical applications of DL in various medical disciplines. In a few radiology applications, DL has demonstrated promising results, but the technology is not yet developed enough to replace the diagnostic occupation of a radiologist [23]. There is a possibility that DL algorithms and radiologists will collaborate to enhance diagnostic effectiveness and efficiency. Numerous studies have investigated the capability of MRI to identify and classify brain tumors utilizing a variety of research methodologies. Afshar et al. developed a modified version of the CapsNet architecture for categorizing the primary brain tumor consisting of 3064 images using tumor boundaries as supplementary inputs to increase effort, surpass previous techniques, and achieve a classification rate of 90.89% [24]. Gumaei et al. proposed a brain tumor classification method using hybrid feature extraction techniques and RELM. The authors preprocessed brain images using min–max normalization, extracted features using the hybrid method, classified them using RELM, and achieved a maximum accuracy of 94.23% [25].

Kaplan et al. proposed brain tumor classification models using nLBP and αLBP feature extraction methods. These models accurately classified the most common brain tumor types, including glioma, meningioma, and pituitary tumors, and achieved a high accuracy of 95.56% using the nLBPD = 1 feature extraction method and KNN model [19]. Rezaei et al. developed an integrated approach for segmenting and classifying brain tumors in MRI images. The methods included noise removal, SVM-based segmentation, feature extraction, and selection using DE. Tumor slices were classified using KNN, WSVM, and HIK-SVM classifiers. Combined with MODE-based ensemble techniques, these classifiers achieved a 92.46% accuracy rate [26]. Fouad et al. developed a brain tumor classification method using HDWT-HOG feature descriptors and the WOA for feature reduction. The approach utilized the Bagging ensemble techniques and achieved an average accuracy of 96.4% with Bagging, and, when used, Boosting attained 95.8% [27].

Ayadi et al. presented brain tumor classification techniques using normalization, dense speeded-up robust features, and the histogram of gradient approaches to enhance the image quality and generate a discriminative feature. In addition, they used SVM for classification and achieved a 90.27% accuracy on the benchmarked dataset [28]. Srujan et al. built a DL system with sixteen layers of CNN to classify the tumor types by leveraging activation functions like ReLU and Adam optimizer, and the system achieved a 95.36% accuracy [29]. Tejaswini et al. proposed a CNN model to detect meningioma, glioma, and pituitary brain tumors with an average training accuracy of 92.79% and validation accuracy of 87.16%; in addition, the tumor region segmentation was performed using Otsu thresholding, Fuzzy c-means, and watershed techniques [30]. Huang et al. developed a CNNBCN to classify brain tumors. The network structure was generated using a random graph algorithm, achieving an accuracy of 95.49% [31].

Ghassemi et al. suggested a DL framework for brain tumor classification. The authors used pre-trained networks as GAN discriminators to extract robust features and learn MR image structures. By replacing the fully connected layers and incorporating techniques like data augmentation and dropout, the method achieved a 95.6% accuracy using fivefold cross-validation [32]. Deepak et al. combined the CNN feature with SVM for the medical image classification of brain tumors. The automated system achieved an accuracy of 95.82% evaluated on the fivefold cross-validation procedure, outperforming the state-of-the-art method [33]. Noreen et al. adapted fine-tuned pre-trained networks, such as InceptionV3 and Xception, for identifying brain tumors. The models were integrated with various ML methods, namely Softmax, SVM, Random Forest, and KNN, and achieved a 94.34% accuracy with the InceptionV3 ensemble [34]. Shaik et al. addressed the challenging task of brain tumor classification in medical image analysis. The authors introduced a multi-level attention mechanism, MANet, which combined spatial and cross-channel attention to prioritize tumors and maintain cross-channel temporal dependencies. The method achieved a 96.51% accuracy for primary brain tumor classification [35].

Ahmad et al. proposed a deep generative neural network for brain tumor classification. The method combined variational auto encoders and generative adversarial networks to generate realistic brain tumor MRI images and achieved an accuracy of 96.25% [36]. Alanazi et al. proposed a deep transfer learning model for the early diagnosis of brain tumor subtypes. The method involved constructing isolated CNN models and adjusting the weights of a 22-layer CNN model using transfer learning. The developed model obtained 95.75- and 96.89-percent accuracies on MRI images [37]. Almalki et al. used an ML approach with MRI to promptly diagnose brain tumor severity (glioma, meningioma, pituitary, and no tumor). They extracted Gaussian and nonlinear scale features, capturing small details by breaking MRIs into 8 × 8-pixel images. The strongest features were selected and segmented into 400 Gaussian and 400 nonlinear scale features, and they were hybridized with each MRI. They obtained a 95.33% accuracy using the SVM classifier [38]. Kumar et al. compared three CNN models (AlexNet, ResNet50, and InceptionV3) to classify the primary tumor types and employed data augmentation techniques. The results showed that AlexNet achieved an accuracy of 96.2%, surpassing the other models [39].

Swati et al. employed a pre-trained deep CNN model and proposed a block-wise fine-tuning technique using transfer learning. This approach was evaluated using a standardized dataset consisting of T1-weighted images. Using minimal preprocessing techniques and excluding handcrafted features, the strategy demonstrated an accuracy of 94.82% with VGG19, VGG16 achieved 94.65%, and AlexNet achieved 89.95% when evaluated using a fivefold cross-validation methodology [40]. Ekong et al. integrated depth-wise separable convolutions with Bayesian techniques to precisely classify and predict brain cancers. The recommended technique demonstrated superior performance compared to existing methods in terms of an accuracy of 94.32% [41].

Asiri et al. enhanced computer-aided systems and facilitated physician learning using artificially generated medical imaging data. A deep learning technique, a Generative Adversarial Network (GAN), was employed, wherein a generator and a discriminator engage in a competitive process to generate precise MRI data. The proposed methodology demonstrated a notable level of precision, with an accuracy rate of 96%. The evaluation of this approach was conducted using a dataset comprising MRI scans collected from various Chinese hospitals throughout the period spanning from 2005 to 2020 [42]. Shilaskar et al. proposed a system comprising three main components: preprocessing, HOG for feature extraction, and classification. The results indicated varying levels of accuracy when employing multiple machine learning classifiers, including SVM, Gradient Boosting, KNN, XG Boost, and Logistic Regression, with the XG Boost classifier attaining the highest accuracy rate of 92.02% [43].

## 3. Materials and Methods

**Figure 1:**
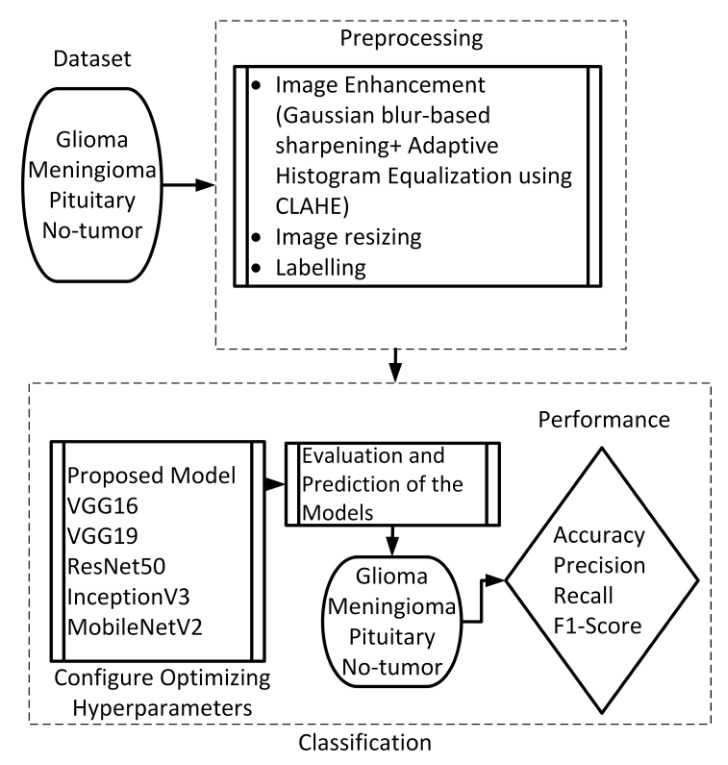
Flow chart of the suggested scheme.

**Figure 2:**
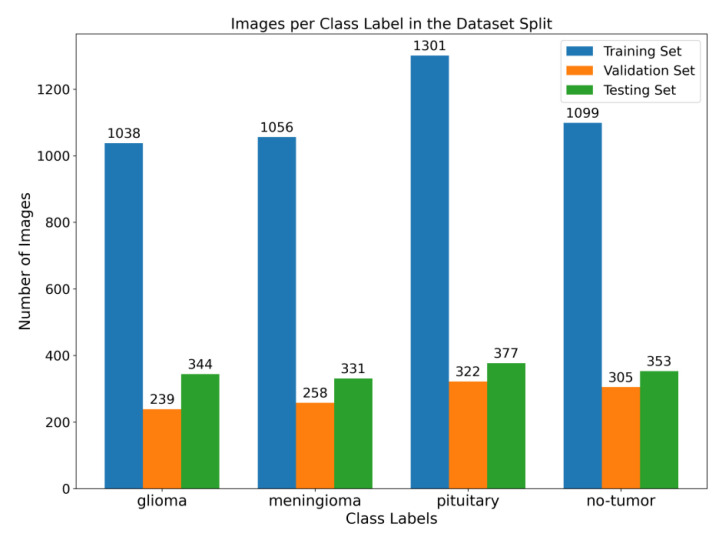
Illustration of the distribution of images among various class labels throughout the training, validation, and testing dataset splits. The bar graph displays the distribution of images across different classes, with the training set at 64%, the validation set at 16%, and the testing set at 20%.

This section presents the proposed method, which consists of two primary components: image preprocessing and model training. The flowchart illustrating the suggested system is presented in Figure 1. To enhance the quality of the image, the preprocessing stage incorporated Gaussian-blur-based sharpening and Adaptive Histogram Equalization techniques using CLAHE. Subsequently, labeled images were resized while maintaining the aspect ratio, normalized, and divided into three sets, as shown in Figure 2. Furthermore, the model underwent training using 5-fold cross-validation [44] using the Adam optimizer and incorporated the ReduceLROnPlateau callbacks to dynamically regulate the learning rate throughout the training process. The effectiveness of the proposed model was evaluated using metrics such as accuracy, precision, recall, and F1-score.

This study employed a publicly accessible MRI dataset Msoud [45], obtained from the Kaggle repository. This dataset combines three publicly accessible datasets, including Figshare [46], SARTAJ [47], and BR35H [48]. It consists of 7023 MRIs of the human brain provided in grayscale and jpg format. The dataset includes primary types of brain tumors, namely glioma, meningioma, pituitary tumors, and images without tumors.

### 3.1. Preprocessing

We implemented a preprocessing framework to improve image quality by integrating sharpening and Contrast-Limited Adaptive Histogram Equalization (CLAHE) approaches. The process of sharpening commenced by implementing a Gaussian blur through the utilization of a specific technique. The utilization of a 5 × 5 kernel was suitable in the process of attenuating high-frequency noise. The resultant enhanced image was determined using the formula:

(1) Sharpened Image=1.5×Original Image−0.5×Blurred Image

Subsequently, the image underwent a conversion process to grayscale, facilitating a precise enhancement of contrast. To achieve this, CLAHE was utilized, characterized by an 8 × 8-tile grid and a clip limit of 2.0. Distinct from global histogram equalization, CLAHE adopts a localized strategy by partitioning the image into discrete tiles and performing individual equalizations, encapsulated by

(2) $$H_{local}(i)=CLAHE(H_{tile}(i))$$

**Figure 3:**
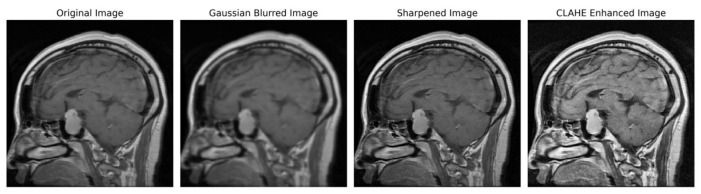
Sequential image improvement as part of the preprocessing framework. The stages progress from the unaltered original image through Gaussian blurring for noise suppression, sharpening the emphasized edge definition to the final enhancement using CLAHE.

In order to ensure accordance with the specifications of the subsequent deep learning framework, the enhanced grayscale image was transformed into the RGB color space [49,50]. Figure 3 illustrates the several stages of enhancing picture quality, from the initial image to the CLAHE-enhanced image. This depiction showcases the effectiveness of our preprocessing method and its notable impact on improving the overall quality of the image.

### 3.2. Proposed Architecture

**Figure 4:**
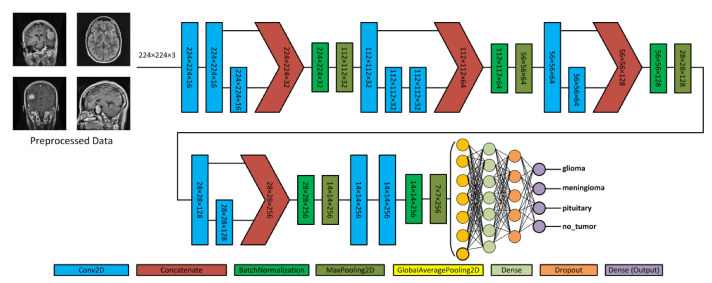
Illustration of the proposed architecture and various forms of brain tumors.

Figure 4 depicts the proposed model, which acquires MRI data with input dimensions of 224 × 224 and reveals its operational characteristics. The model consists of multiple server blocks. A convolutional layer [51] was employed in the initial stage, consisting of 16 filters. Each filter was employed with a kernel size of 3 × 3 and a stride size of 1 × 1. A normalizing layer [52] and a 2D (two-dimensional) max pooling layer with a size of 2 × 2 were employed to maximize the information among the intermediate layer’s output. Similarly, we integrated additional convolutional layers into the model, utilizing 32, 64, 128, and 256 filter sizes. Each filter utilized in this study had a kernel size of 3 × 3 and a stride size of 1 × 1, and the same and valid padding was suitable for the experiment. As illustrated in Figure 4, skip connections were employed within each block to facilitate the information flow by concatenating the outputs of specific convolutional layers. Subsequently, a dense layer of 512 neurons was employed, accompanied by global average pooling and activation through the rectified linear unit (ReLU) function.

To mitigate the issue of overfitting, the dense layer was subjected to regulation using L1 (10^−5^) and L2 (10^−4^) regularization techniques [53]. During the training process, the neurons within a dropout layer [54] were randomly deactivated at a rate of 0.5% to enhance regularization implementation further. Finally, the output layer employed the softmax algorithm [51] to compute the probability score for each class and classify whether the input image exhibited a glioma, meningioma, pituitary, or no tumor. In addition, the model employed the Adam optimizer [55,56], categorical cross-entropy for loss functions, and the ReduceLROnPlateau callback to optimize the learning rate [57]. The model was trained with a batch size of 8 for 30 epochs.

Convolutional neural networks are widely used for image classification tasks. In the proposed model, 2D convolution involved applying a kernel to the input data to extract features. The convolution operation captures spatial dependencies and hierarchies within the data. The convolution operation in a 2D CNN can be mathematically defined as follows:

(3) $$Y_{ij}=\sum_{m}\sum_{n}X_{\left(i+m)(j+n\right)}.\;K_{mn}$$

where $Y_{ij}$ represents the output element at the position *i*, *j*; $X_{\left(i+m)(j+n\right)}$ denotes the input elements at the position (*i* + *m*, *j* + *n*); and $K_{\left(mn\right)}$ signifies the kernel element at the position (*m*, *n*). The equation involves summing the element-wise multiplication of the input element and corresponding kernel element across the indices *m* and *n*. This operation is applied across the entire input to compute the element of the output feature map. The convolution operation efficiently captures local patterns and interactions between neighboring elements, enabling the network to learn the hierarchical representation and extract meaningful features from the input data. Furthermore, the convolutional operation involved applying the kernel to input using a sliding window. The kernel size determines the local region considered, and the stride size controls the movement of the kernel. Padding preserves spatial dimensions. The output size can be calculated using the following equation.

(4) $$O=\left\lfloor \frac{I-K+2P}{S}\right\rfloor +1$$

where *O* represents the output size, *I* denotes the input size, *K* represents the kernel size, *S* denotes the stride size, and *P* represents the padding size [51].

#### 3.2.1. Batch Normalization

Batch normalization (BN) is used in deep neural networks to normalize the intermediate layers’ outputs. It suits internal covariate shifts, improving training, stability, and performance. In our proposed model, we incorporated the BN layer, following the skip connections and preceding the Max Pooling layer. The rationale behind this design was attributed to the function of skip connections, which involves the concatenation of feature maps originating from distinct layers. Including the BN layer immediately after ensures that the aggregated feature maps undergo normalization, preserving a uniform scale and distribution before pooling. In addition to normalization, the positioning of BN also provides regularization, hence mitigating the potential issue of overfitting and ensuring that the pooling layer functions on standardized activations. The equation can represent the normalization process.

(5) $$y=\frac{x-\mu }{\sigma }.\gamma +\beta$$

where *x* is the input; µ and σ; are the mean and standard deviation computed over a mini-batch size, respectively; and γ and β are learnable scaling and shifting parameters, respectively.

#### 3.2.2. Pooling Layers

The pooling operation is used in a CNN for downsampling, and the input feature map is divided into non-overlapping regions or pooling windows. The purpose is to calculate the maximum value of each window, resulting in a downscaled output feature map. The following equation represents the max pooling operation at the position (i,j) in the output feature map.

(6) Maxpooling(x)(i,j)=(∀m,n)max(x)(i+m,j+n)

Max pooling (x)(i,j) denotes the value at the position (i,j) in the output feature map after max pooling. The term ∀m,n represents the double summation over the indices m and n and covers all possible values within the pooling windows. max(x)(i+m,j+n) represents the maximum value among the neighboring elements in the input feature map, specifically at positions (i+m,j+n). The global average pooling (GAP) operation reduces the spatial dimension of a feature map while capturing the average representation of the entire feature map. The GAP can be formulated as follows:

(7) $$GobalAvgPooling(x)=\frac{1}{k\times 1}\sum_{i=1}^{k}\sum_{j=1}^{l}x_{i,j}$$

The equation illustrates the operational mechanism of GAP applied to a feature map (*x*). The feature map is characterized by l dimensions for height, width, and channels (*k*). The symbol ∑ denotes the mathematical operation of summation and the variables *i* and *j* are employed to iterate through the spatial dimensions of the feature map. The *k* values in the resulting vector correspond to the mean activation of the relevant channel across all spatial positions in the feature map [53].

#### 3.2.3. Activation and Loss Functions

ReLU is an activation function that introduces nonlinearity into a neural network [58]. It takes an input value and returns the maximum value and 0. Mathematically the ReLU function can be defined as

(8) ReLU(x)=max(0,x)

where x is the input value; if the input value is positive, *ReLU* outputs the same value. If the input value is negative, *ReLU* outputs 0.

The utilization of the softmax function occurs in the output layer of the proposed model planned for multi-classification tasks. The process converts a vector of real input values into a probability distribution across different classes. The mathematical expression for the softmax role is as follows:

(9) $$Softmax(x_{i})=\frac{exp(x_{i})}{\sum_{j=1}^{4}exp(x_{j})},for\;i=1,2,3,4$$

The equation $x_{i}$ represents the *i*-th element of the input vector, and the softmax function normalizes each probability by dividing it by the sum of the exponential value of all probabilities in the vector. Furthermore, the loss function was utilized to measure the discrepancy between the algorithm’s predictions and actual values. Various optimization techniques can be applied to minimize this error. In addition, categorical cross-entropy was chosen as the loss function. Categorical cross-entropy can be calculated as the error rate using the equation.

(10) $$Categorical\;Cross\;Entropy=-\sum_{i}^{N}y_{true}\left[i\right].log(y_{pred}\left[i\right])$$

where N is the number of classes, $y_{true}\left[i\right]$ represents the true class probabilities, and $y_{pred}\left[i\right]$ denotes the predicted probabilities of each class.

#### 3.2.4. Optimization Techniques

**Figure 5:**
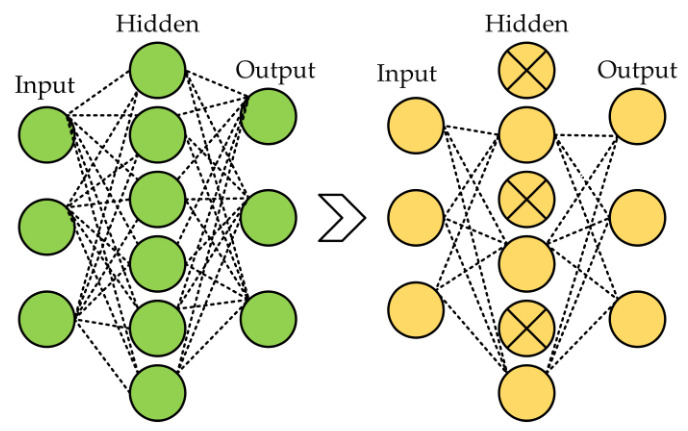
The right side of the diagram visually depicts a dropout layer characterized by a dropout rate of 50%.

Several regularization strategies were used in the proposed model, including dropout, L1, L2, and ReduceLROnPlateau callbacks to reduce the overfitting in neural networks. Dropout arbitrarily changes a small portion of the input units (neurons) to zero during the training phase [59]. By preventing the network from being overly dependent on particular units and encouraging generalization, this dropout process aids in the network learning redundant representations. The model becomes more resilient and enhances its capacity to perform effectively on unknown data by injecting this unpredictability through the 50% dropout rate, thereby improving its overall performance. The 50% dropout example is shown in Figure 5.

L1 and L2 strategies are employed in the neural network to mitigate the issue of overfitting and enhance the accuracy when activated with novel data from the problem domain [60]. These techniques were employed in the proposed model due to their effectiveness among the standard regularization methods. L1 regularization is also known as Lasso regression, and L2 regularization is known as weight decay or ridge regression. The cost drives for L1 and L2 can be defined as follows:

(11) $$\begin{array}{l} L1Regularization(LassoRegression):\\ Cost\;Function=Loss\;Funtion+\lambda \sum_{i=1}^{N}\left|w_{i}\right|\\ L2Regularization(Weight\;Decay\;or\;RidgeRegression):\\ Cost\;Function=Loss\;Funtion+\lambda \sum_{i=1}^{N}\left|w_{i}^{2}\right| \end{array}$$

where λ is the hyperparameter that regulates the strength of regularization, *N* is denoted as the model factors, $w_{i}$ embodies *i*-th parameters, and ∑ denotes the sum of all parameters. The cost function combines the loss, representing the error between predicted and target values, with a regularization term to form the overall objective function.

In the proposed model, we utilized the ReduceLROnPlateau from Keras [61]. This callback is crucial in reducing the learning rate (LR) during the model training phase, specifically when validation losses showed no further improvement. Incorporating this callback enabled the optimization process to take smaller steps toward minimizing the loss function, resulting in a more efficient model. During the training phase, the ReduceLROnPlateau callback monitored the chosen metric, such as validation loss. The system recorded the optimal observed value for this metric and assessed whether the current value demonstrated improvement over a predetermined number of epochs. If the monitored metric did not exhibit improvement, the callback triggered a reduction in the learning rate. We employed a factor that was set while configuring the ReduceLROnPlateau callbacks to achieve the learning rate reduction. In the proposed model, we initially set the learning rate to 0.001 and utilized a reduction factor (F) of 0.4; the new learning rate (New LR) can be calculated by applying the given equation.

(12) New LR=LR×F

### 3.3. Pre-Trained Model

Pre-trained neural networks are ML models that have undergone training on extensive datasets like ImageNet, consisting of various images belonging to various classes. Pre-trained models have proven highly advantageous in various tasks, including image classification and object detection. Pre-trained models are employed because of their ability to graph data patterns, allowing them to be used as a starting point for new tasks without having to start the training process from scratch. This investigation included five pre-trained models, namely VGG16, ResNet50, MobileNetV2, InceptionV3, and VGG19.

#### 3.3.1. VGG16

The VGG16 model was initially presented in 2014 by Simonyan and Zisserman [62], scholars affiliated with the Visual Geometry Group at the University of Oxford. The architectural design incorporates filters of dimensions 3 × 3, a stride of 1, and 16 layers, consisting of three fully connected layers and thirteen convolutional layers. The maximum pooling layers employ pooling windows with dimensions of 2 by 2 and a stride of 2. VGG16, a widely recognized choice for efficient feature extraction in transfer learning, boasts a substantial parameter count of 138 million.

#### 3.3.2. ResNet50

Deep neural networks demonstrate improved performance as their depth increases, as evidenced in the literature [63]. The challenges related to this improvement arise from vanishing or exploding gradients, manifesting as the neural network expands. To overcome this impediment, the authors of [64] have proposed ResNet50, an innovative approach that utilizes residual modules to facilitate the learning of residual mapping instead of conventional input–output mapping. This innovative approach involves incorporating the input into the output of the modules through shortcut connections that circumvent certain levels. Consequently, including residual blocks effectively mitigates the problem of vanishing gradients, thereby preventing a decline in performance as the network depth increases. The ResNet50 architecture incorporates convolutional layers of varying filter sizes (1 × 1, 3 × 3, 1 × 1) within bottleneck blocks interspersed with max pooling and average pooling layers to facilitate extracting features from the input.

#### 3.3.3. MobileNetV2

The architectural design aims to provide mobile and embedded applications, achieving a remarkable balance between high accuracy, lightweight computation, and optimal memory usage. The employed model utilized three primary strategies: the inverted residual, the linear bottleneck, and the width multiplier parameters. Using convolutional layers in the inverted residual technique increases network capacity while concurrently reducing the computational requirements and memory usage. The input is improved by increasing the number of channels and applying convolution using a small kernel size to achieve this objective. Subsequently, the resulting output is projected onto a reduced number of channels. In contrast, linear bottlenecks employ a linear activation function instead of a non-linear one, aiming to minimize the number of parameters needed. Furthermore, utilizing width multiplier parameters can adjust the number of channels within a network, thereby introducing enhanced adaptability [65].

#### 3.3.4. InceptionV3

The InceptionV3 architecture is a CNN that belongs to the inception series. It is recognized for its significant advancements compared to previous iterations. The proposed approach employs an advanced design strategy wherein the network’s capacity is expanded by incorporating multiple kernel sizes at a given level instead of increasing depth through stacked layers. The proposed methodology employs inception modules, which integrate a max pooling layer with varying kernel sizes of 1 × 1, 3 × 3, and 5 × 5 to effectively capture a wide range of features at different scales. The resulting output is obtained by concatenating the outputs of these layers, which is achieved by including a 1 × 1 convolution layer before the 3 × 3 and 5 × 5 convolutional layers. This additional layer decreases the number of input channels and optimizes the utilization of computational resources [66].

#### 3.3.5. VGG19

The VGG19 architecture modified the VGG16 architecture, encompassing nineteen layers. This included sixteen convolutional layers, three fully connected layers, a compact filter with dimensions of 3 × 3, and a stride size 1. Additionally, the model incorporated max pooling layers that employ a pooling of size 2 × 2 and a stride size of 2. With a parameter count of 144 million, this model surpasses VGG16 in terms of power, although at the cost of increased computational requirements [62].

## 4. Experimental Results

This study employed the proposed model to categorize a substantial MRI dataset comprising 7023 images. The dataset encompassed glioma, meningioma, pituitary cases, and cases with no tumor. Initially, a preprocessing stage was incorporated to enhance the feature extraction. In this stage, image enhancement techniques with Gaussian blur and CLAHE were applied to improve the quality of the images. The dataset was divided into subsets, namely training, validation, and testing. The dataset was trained using the Adam optimizer and subsequently assessed through a fivefold cross-validation method. Algorithm 1 presents the procedure for the training and evaluation process.
**Algorithm 1:** Training and Evaluation Process with 5-fold Cross-Validation*1. Initialize Metrics List*  *. final_test_metrics = []* *2. Combine Training and Validation sets*  *. S = N train + N val        where S represents the dataset* *3. 5-Fold Cross - Validation*  *. For i in {1, 2, 3, 4, 5}:*    *3.1. Data Splitting*      $\;.\;{Train}_{i}$$=S\;–\;S_{i}$      $\;.\;{Val}_{i}$$={\;S}_{i}$    *3.2. Train Model*       *.Train the model on* ${Train}_{i}$ *and validate on* ${Val}_{i}$      .*Setup Callbacks and Optimizer*    *3.3. Evaluate on Test set (T) where T represents the testing data*      *.temp_metrics = Model. Evaluate (T)*      *.Append temp_metrics to final_test_metrics*   *4. Calculate Average Test Metrics*     *.Metrics final =*
$\frac{1}{5}\sum_{i=1}^{5}final\_test\_metrics[i]$ *5. Output*  *. Metrics final contains the average values on the set T*

**Figure 6:**
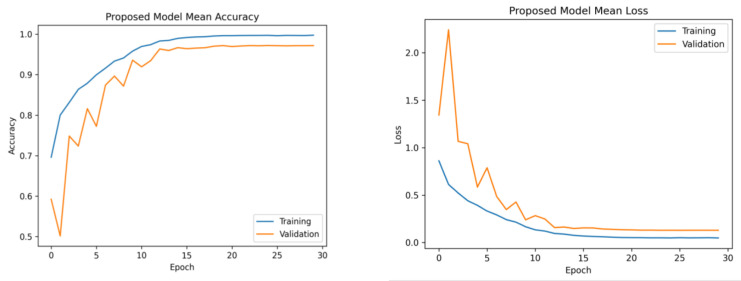
Mean accuracy and losses of the proposed model during 5-fold cross-validation. (**Left**): mean accuracy progression across training folds. (**Right**): corresponding mean loss trend. This demonstrates consistent accuracy improvement and decreasing loss, highlighting effective model training.

The learning rates were optimized using the ReduceLROnPlateau callbacks, and a batch size of 8 was utilized. Figure 6 presents the average accuracy and losses of the model proposed in this study. During the initial stage of training, the graphs display fluctuations, which can be attributed to the utilization of the ReduceLROnPlateau callback. The primary objective of this callback is to dynamically modify the learning rate of the optimizer during the training process, specifically when the loss function reaches a plateau. After completing 12 epochs, the optimizer demonstrates a gradual convergence toward an optimal configuration of weights, resulting in diminished fluctuations observed in the accuracy and loss curves.

Furthermore, the platform utilized several libraries, such as Numpy, Pandas, Matplotlib, Sklearn, Keras, and TensorFlow, to enhance the efficiency of data processing and model development. The computation was performed on an Intel Core i7-7800 CPU operating at a clock speed of 3.5 GHz. The model training and tuning were managed using an NVIDIA GeForce GTX 1080 Ti GPU. The selection of Python 3.7 as the primary programming language for this study was based on its comprehensive set of tools for data manipulation, analysis, and visualization. The platform successfully preserved the data employed in this study due to its substantial RAM capacity of 32 GB.

### Model Evaluation Matrices

The suggested framework was subjected to a thorough evaluation, which involved an analysis of its precision, recall, F1-score, and accuracy. Precision evaluates the model’s ability to minimize the misclassification of negative examples as positive, and the term “is derived from” refers to the calculation of a specific metric, which is obtained by dividing the number of true positives by the sum of true positives and false positives. However, it is important to note that recall is a metric that measures the model’s capacity to classify the appropriate tumor type accurately. This is calculated by dividing the number of true positives by the sum of true positives and false negatives. The F1-score is a metric used in evaluation that quantifies the balance between precision and recall. It is calculated as the harmonic mean of precision and recall, obtained by multiplying precision and recall and dividing the result by their sum, multiplied by two. In the context of classification models, accuracy measures the model’s overall performance by quantifying the proportion of correct classifications. It is calculated by dividing the number of accurate predictions by the total number of predictions made. Equations (13)–(16) indicate the mathematical representations of precision, recall, F1-score, and accuracy [67].

(13) $$Precision=\frac{TP}{TP+FP}$$

(14) $$Recall=\frac{TP}{TP+FN}$$

(15) $$F1-Score=2\times \frac{Recall\times Precision}{Recall+Precision}$$

(16) $$Accuracy=\frac{TP+TN}{TP+TN+FP+FN}$$

The evaluation results, including the average precision, recall, F1-score, and accuracy for both the proposed and pre-trained models, are presented in Table 1. The suggested framework demonstrated a notable accuracy rate of 97.84%. Moreover, it achieved precision and recall values of 97.85% and an F1-score of 97.90%. On the contrary, the InceptionV3 model exhibited the lowest performance, achieving an accuracy of 88.15%, a precision rate of 87.70%, a recall rate of 87.89%, and an F1-score rate of 87.60%. The observed variation in the performance of InceptionV3 can be ascribed to its utilization of multiple and parallel modules, which may not be well suited for the specific characteristics of this dataset, as supported by our research findings. The pre-trained models VGG16, ResNet50, and VGG19 exhibited superior performance compared to MobileNetV2. Furthermore, the pre-trained models employed the standard input dimensions, including VGG16, VGG19, ResNet50, and MobileNetV2 with dimensions of 224 × 224 and InceptionV3 with dimensions of 299 × 229. In order to preserve the pre-existing weights, the layers of the base model were designated as non-trainable.

**Figure 7:**
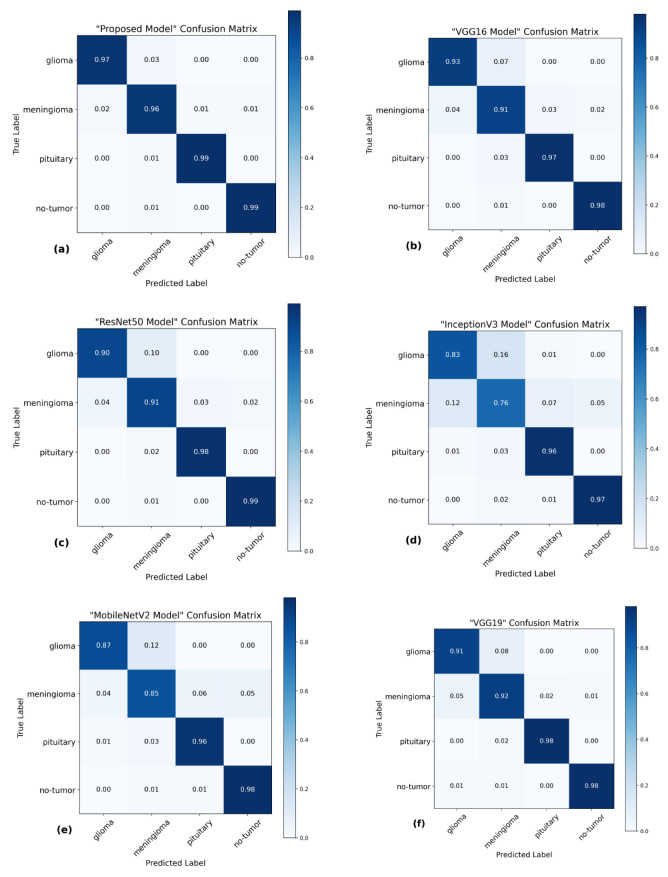
Confusion matrices of several models using the testing data. (**a**) The proposed model has a high level of accuracy, achieving a score of 97.84%. (**b**) VGG16 model achieved a classification accuracy of 95.00%. (**c**) ResNet50 model achieved an accuracy of 94.75%. (**d**) The accuracy of InceptionV3 is 88.15%. (**e**) MobileNetV2 model achieved a classification accuracy of 91.73%. (**f**) VGG19 model achieved a classification accuracy of 94.83%.

The utilization of the confusion matrix is a fundamental assessment instrument for classification models [68]. The proposed network demonstrated robust capabilities in accurately classifying various types of brain tumors, effectively identifying each type during the examination. Figure 7 presents a visual representation of the results obtained from the testing data, enabling a comparison between the proposed and pre-trained models. The comparison reveals that the proposed model outperformed the pre-trained models in performance. The proposed model demonstrated high accuracy in predicting glioma, achieving 97%, and meningioma, achieving a 96% accuracy rate. Additionally, it achieved a 99% accuracy rate in predicting pituitary and no-tumor cases. These results surpass the performance of pre-trained models. However, it is crucial to emphasize that the efficacy of treatment for glioma and meningioma in this study did not achieve comparable levels of success. This finding underscores the necessity for additional research and investigation in subsequent studies.

Furthermore, the Receiver Operating Characteristics (ROC) curve is a visual representation of the performance of a classification model across different classification thresholds [69]. The True Positive Rate (TPR) and False Positive Rate (FPR) are graphically represented. The ROC curve illustrates the balance between correctly identifying positive and incorrectly classifying negative instances as positive at all classification thresholds on the testing set. The ROC curve provides insights into the model’s ability to differentiate between different thresholds effectively.

The present investigation demonstrates the proposed framework’s superior diagnostic efficacy compared to pre-trained designs. The findings of this study provide evidence supporting the suggested model’s higher diagnostic accuracy compared to state-of-the-art methodologies. When comparing the performance of the VGG16 architecture, it was observed that it achieved scores of 0.95 for glioma, 0.93 for meningioma, 0.97 for pituitary, and 0.98 for the no-tumor category. The ResNet50 architecture achieved classification scores of 0.92, 0.93, 0.97, and 0.98 for the glioma, meningioma, pituitary, and no-tumor classes, respectively. The InceptionV3 model yielded predictive scores of 0.84 for glioma, 0.81 for meningioma, 0.96 for pituitary, and 0.97 for the no-tumor category. The MobileNetV2 design achieved scores of 0.90, 0.86, 0.97, and 0.98 for the glioma, meningioma, pituitary, and no-tumor categories, respectively. Additionally, the VGG19 architecture demonstrated classification scores of 0.92 for glioma, 0.93 for meningioma, 0.98 for the pituitary, and 0.98 for the no-tumor category.

**Figure 8:**
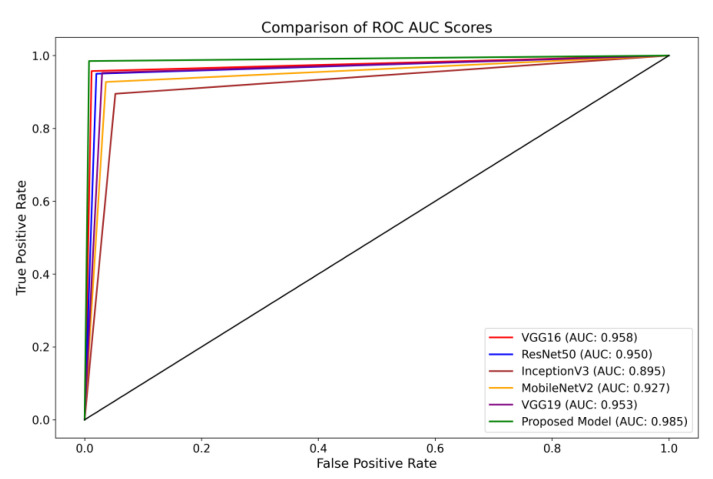
Illustration of a comprehensive visual representation that compares the proposed model’s overall ROC score with other pre-trained models.

The model under consideration demonstrates notable performance regarding ROC scores. The achieved classification accuracies are as follows: 0.98 for glioma, 0.97 for meningioma, 0.99 for pituitary, and a flawless accuracy of 1.00 for the no-tumor category. The robust performance of the model is supported by a collective ROC score of 98.50%, as depicted in Figure 8, compared to pre-trained models.

## 5. Discussion

This investigation introduces a novel methodology for categorizing the Msoud dataset, which consists of a varied assortment of 7023 brain images. The efficacy of the proposed system is demonstrated by its capacity to attain highly precise prediction outcomes, surpassing prior research endeavors with comparable aims. Moreover, this study proposes a method that does not rely on segmenting brain tumor images for classification purposes. The primary advantage of our approach resides in its capacity to substantially diminish the requirement for manual procedures, such as feature extraction and tumor localization. These processes are not only time-intensive but also susceptible to inaccuracies. By employing various enhancement techniques, including sharpening with Gaussian blur and Contrast-Limited Adaptive Histogram Equalization (CLAHE), notable enhancements are achieved in the quality of the brain images. The enhancement process plays a crucial role in the refinement of edges and improving the overall image clarity, reducing the manual effort needed for feature extraction.

Furthermore, our proposed model incorporates distinctive concatenation concepts within the convolutional layers, demonstrating superior performance compared to alternative methods, as shown in Table 2. By incorporating these enhancement techniques, the proposed model has demonstrated exceptional performance, surpassing the existing state-of-the-art model in classifying brain tumors. The successful accomplishment is evidence of the proposed model’s resilience and capacity to apply to a wide range of brain image classification tasks, highlighting its potential for achieving precise and dependable results. Integrating decreased manual intervention, enhanced image quality, and the suggested model architecture renders our approach highly promising for practical implementations in classifying brain tumors.

The methodology of Gumaei et al. [25] introduced a combination of PCA, NGIST, and RELM. While this hybrid approach attempted to capture a comprehensive feature set, PCA might not always capture non-linear patterns inherent in brain images, potentially missing crucial tumor-specific details and resulting in less accuracy. The methodologies of Swati et al. [40] and Noreen et al. [34] relied on refining generic architectures, specifically state-of-the-art models. Such fine-tuning of deep architectures can be resource-intensive. The intricate process necessitates substantial computational resources and proves time-consuming, given the need to adjust many parameters in these extensive networks. Contrarily, our model is purposefully designed for brain tumor classification. It captures tumor-specific attributes efficiently without the excessive computational demands typically associated with deep architectures. As corroborated by Table 1, our method requires fewer parameters than the state of the art and delivers faster testing times.

Ghassemi et al. [32] ventured into the territory of Generative Adversarial Networks, leveraging CNN-based GANs. While GANs are adept at generating synthetic images, their direct application to classification might introduce synthetic nuances that deviate from real-world MRI variations, potentially affecting classification accuracy. Huang et al. [31] introduced the CNNBCN, a model rooted in randomly generated graph algorithms, achieving an accuracy of 95.49% and demonstrating advancements in neural network design. In contrast, our methodology performs superior classification on extensive tumor and no-tumor images.

Techniques like HDWT-HOG-Bagging and NLBP-αLBP-KNN, as presented by Fouad et al. [27] and Kaplan et al. [19], rely heavily on traditional feature extraction. While computationally intensive, such methods might still miss subtle details and patterns in the MRI scans, resulting in less accuracy. Ayadi et al. [28] employed DSURF-HOG combined with SVM for classification, a method that might overlook hierarchical and spatial patterns in MRI images, which deep learning models can capture more effectively.

Ekong et al. [41] introduced a Bayesian-CNN approach, and while Bayesian methods offer probabilistic insights, they might not always capture the intricate features of brain tumors. While the GAN-Softmax approach by Asiri et al.’s [42] model offers certain advancements, it is computationally more demanding. Moreover, the efficacy of methodologies such as HOG-XG Boost by Shilaskar et al. [43] and the SURF-KAZE technique by Almalki et al. [38] might be constrained, particularly in their ability to capture spatial and hierarchical MRI patterns—areas where contemporary deep learning models exhibit proficiency as proved in this study.

### Limitations

The usefulness of the proposed methodology for extracting features has been proven by using a specific dataset obtained from MRI scans. In order to enhance the clarity of the images, various techniques for image enhancement were employed. Although these strategies can enhance visibility, it is crucial to acknowledge that, in specific circumstances, it may impact classification accuracy. Therefore, comprehensive evaluations are necessary to test the method’s suitability for different imaging modalities and clinical scenarios and its flexibility for image enhancements.

## 6. Conclusions

The present study introduced a novel approach to classify various categories of brain tumors, such as primary, meningioma, pituitary, and instances with no tumor. This is achieved by combining image enhancement techniques, namely, Gaussian-blur-based sharpening and Contrast-Limited Adaptive Histogram Equalization (CLAHE), with a proposed convolutional neural network. The findings of our study demonstrate a remarkable level of accuracy, specifically 97.84%, which was achieved through a diligent evaluation of the effectiveness of the suggested framework. The outcome of this study showcases the model’s robust capacity for generalization, rendering it a valuable and dependable tool within the medical field. The capacity of this method to facilitate expeditious and accurate decision making by medical professionals in the realm of brain tumor diagnosis is evident. To enhance patient care in the future, we intend to revolutionize medical imaging methods. This will be accomplished by creating real-time brain tumor detection systems and establishing three-dimensional networks to analyze other medical images.

## Figures and Tables

**Table 1:** Evaluation results of proposed and pre-trained models.

Models Name	Total Params:	Precision Average (%)	Recall Average (%)	F1-Score Average (%)	Accuracy Average (%)	Testing Time Average (s)
VGG16	14,979,396	95.00	94.85	94.90	95.00	2.29
ResNet50	24,638,852	94.59	94.64	94.55	94.75	1.91
InceptionV3	55,362,340	87.70	87.89	87.60	88.15	2.61
MobileNetV2	2,915,908	91.65	91.40	91.60	91.73	0.99
VGG19	20,289,092	94.80	94.65	94.70	94.83	2.64
Proposed Model	1,708,356	97.85	97.85	97.90	97.84	0.83

**Table 2:** Comprehensive comparison of the obtained and previous studies’ results.

Authors	Year	Methods	Dataset	Classes	Precision	Recall	F1-Score	Accuracy
Gumaei et al. [25]	2019	Hybrid PCA-NGIST-RELM	Figshare 3064 Images	3	X	X	X	94.23
Swati et al. [40]	2019	VGG16 Fine tune	Figshare 3064 Images	3	89.17	X	91.50	94.65
Swati et al. [40]	2019	VGG19 Fine Tune	Figshare 3064 Images	3	89.52	X	91.73	94.82
Ghassemi et al. [32]	2019	CNN-based GAN	Figshare 3064 Images	3	95.29	X	95.10	95.60
Huang et al. [31]	2020	CNNBCN	Figshare 3064 Images	3	X	X	X	95.49
Fouad et al. [27]	2020	HDWT-HOG-Bagging	Figshare 3064 Images	3	X	X	X	96.40
Kaplan et al. [19]	2020	NLBP-αLBP-KNN	Figshare 3064 Images	3	X	X	X	95.56
Ayadi et al. [28]	2020	DSURF-HOG -SVM	Figshare 3064 Images	3	X	88.84	89.37	90.27
Noreen et al. [34]	2021	InceptionV3 Ensemble	Figshare 3064 Images	3	93.00	92.00	92.00	94.34
Almalki et al. [38]	2022	SURF-KAZE-SVM	Kaggle 2870 Images	4	X	X	X	95.33
Ekong et al. [41]	2022	Bayesian-CNN	Benchmark BRATS 2015 4000 Images	4	94	95	94	94.32
Asiri et al. [42]	2023	GAN-Softmax	Kaggle 2870 Images	4	92	93	93	96.00
Shilaskar et al. [43]	2023	HOG-XG Boost	Figshare, SARTAJ and Br35H 7023 images	4	92.07	91.82	91.85	92.02
Our work	-	Image Enhancement + Proposed Model	Figshare, SARTAJ and Br35H 7023 images	4	97.85	97.85	97.90	97.84

## Data Availability

The data will be available on reasonable request from the corresponding author.

## Abbreviations

DE	Differential Evolution	AI	Artificial Intelligence
SVM	Support Vector Machine	KNN	K-Nearest Neighbors
WSVM	Weight Kernel Width SVM	DL	Deep learning
HIK-SVM	Histogram Intersection Kernel SVM	ML	Machine learning
HDWT	Haar Discrete Wavelet Transforms	MRI	Magnetic Resonance Imaging
HOG	Histogram of Oriented Gradients	LPB	Local Binary Pattern
MODE	Multi-Objective Differential Evolution	SURF	Speeded Up Robust Feature
GAN	Generative Adversarial Network	WOA	Whale Optimization Algorithm
CNNBCN	Convolutional Neural Network based on Complex Network	PCA	Principal Component Analysis
RELM	Regularized Extreme Learning Machine	CNN	Convolutional Neural Network
CLAHE	Contrast-Limited Adaptive Histogram Equalization	CPU	Central Processing Unit

## References

[1] Khazaei Z., Goodarzi E., Borhaninejad V., Iranmanesh F., Mirshekarpour H., Mirzaei B., Naemi H., Bechashk S.M., Darvishi I., Ershad Sarabi R. (2020). The association between incidence and mortality of brain cancer and human development index (HDI): An ecological study. BMC Public Health.

[2] GLOBOCAN (2020). The Global Cancer Observatory—All Cancers. Int. Agency Res. Cancer—WHO.

[3] Johns Hopkins Medicine Gliomas. https://www.hopkinsmedicine.org/health/conditions-and-diseases/gliomas.

[4] Mayo Clinic Pituitary Tumors—Symptoms and Causes. https://www.mayoclinic.org/diseases-conditions/pituitary-tumors/symptoms-causes/syc-20350548.

[5] Johns Hopkins Medicine Meningioma. https://www.hopkinsmedicine.org/health/conditions-and-diseases/meningioma.

[6] Merck Manuals Consumer Version Overview of Brain Tumors—Brain, Spinal Cord, and Nerve Disorders. https://www.merckmanuals.com/home/brain,-spinal-cord,-and-nerve-disorders/tumors-of-the-nervous-system/overview-of-brain-tumors.

[7] American Brain Tumor Association (2014). American Brain Tumor Association Mood Swings and Cognitive Changes. https://web.archive.org/web/20160802203516/http://www.abta.org/brain-tumor-information/symptoms/mood-swings.html.

[8] Tiwari A., Srivastava S., Pant M. (2020). Brain tumor segmentation and classification from magnetic resonance images: Review of selected methods from 2014 to 2019. Pattern Recognit. Lett..

[9] Iwendi C., Khan S., Anajemba J.H., Mittal M., Alenezi M., Alazab M. (2020). The use of ensemble models for multiple class and binary class classification for improving intrusion detection systems. Sensors.

[10] Ahmad S., Ullah T., Ahmad I., Al-Sharabi A., Ullah K., Khan R.A., Rasheed S., Ullah I., Uddin M.N., Ali M.S. (2022). A Novel Hybrid Deep Learning Model for Metastatic Cancer Detection. Comput. Intell. Neurosci..

[11] Zhuang Y., Chen S., Jiang N., Hu H. (2022). An Effective WSSENet-Based Similarity Retrieval Method of Large Lung CT Image Databases. KSII Trans. Internet Inf. Syst..

[12] Li C., Lin L., Zhang L., Xu R., Chen X., Ji J., Li Y. (2021). Long noncoding RNA p21 enhances autophagy to alleviate endothelial progenitor cells damage and promote endothelial repair in hypertension through SESN2/AMPK/TSC2 pathway. Pharmacol. Res..

[13] Deng X., Liu E., Li S., Duan Y., Xu M. (2023). Interpretable Multi-Modal Image Registration Network Based on Disentangled Convolutional Sparse Coding. IEEE Trans. Image Process..

[14] Zhang K., Yang Y., Ge H., Wang J., Lei X., Chen X., Wan F., Feng H., Tan L. (2022). Neurogenesis and Proliferation of Neural Stem/Progenitor Cells Conferred by Artesunate via FOXO3a/p27Kip1 Axis in Mouse Stroke Model. Mol. Neurobiol..

[15] Wang F., Wang H., Zhou X., Fu R. (2022). A Driving Fatigue Feature Detection Method Based on Multifractal Theory. IEEE Sens. J..

[16] Gao Z., Pan X., Shao J., Jiang X., Su Z., Jin K., Ye J. (2022). Automatic interpretation and clinical evaluation for fundus fluorescein angiography images of diabetic retinopathy patients by deep learning. Br. J. Ophthalmol..

[17] Xu H., Van Der Jeught K., Zhou Z., Zhang L., Yu T., Sun Y., Li Y., Wan C., So K.M., Liu D. (2021). Atractylenolide I enhances responsiveness to immune checkpoint blockade therapy by activating tumor antigen presentation. J. Clin. Investig..

[18] Ao J., Shao X., Liu Z., Liu Q., Xia J., Shi Y., Qi L., Pan J., Ji M. (2023). Stimulated Raman Scattering Microscopy Enables Gleason Scoring of Prostate Core Needle Biopsy by a Convolutional Neural Network. Cancer Res..

[19] Kaplan K., Kaya Y., Kuncan M., Ertunç H.M. (2020). Brain tumor classification using modified local binary patterns (LBP) feature extraction methods. Med. Hypotheses.

[20] Rathi V.G.P., Palani S. (2015). Brain Tumor Detection and Classification Using Deep Learning Classifier on MRI Images. Res. J. Appl. Sci. Eng. Technol..

[21] Cheng J., Huang W., Cao S., Yang R., Yang W., Yun Z., Wang Z., Feng Q. (2015). Enhanced Performance of Brain Tumor Classification via Tumor Region Augmentation and Partition. PLoS ONE.

[22] McBee M.P., Awan O.A., Colucci A.T., Ghobadi C.W., Kadom N., Kansagra A.P., Tridandapani S., Auffermann W.F. (2018). Deep Learning in Radiology. Acad. Radiol..

[23] Lu S., Yang J., Yang B., Yin Z., Liu M., Yin L., Zheng W. (2022). Analysis and Design of Surgical Instrument Localization Algorithm. Comput. Model. Eng. Sci..

[24] Afshar P., Plataniotis K.N., Mohammadi A. Capsule Networks for Brain Tumor Classification Based on MRI Images and Coarse Tumor Boundaries. Proceedings of the ICASSP 2019—2019 IEEE International Conference on Acoustics, Speech and Signal Processing (ICASSP).

[25] Gumaei A., Hassan M.M., Hassan M.R., Alelaiwi A., Fortino G. (2019). A Hybrid Feature Extraction Method with Regularized Extreme Learning Machine for Brain Tumor Classification. IEEE Access.

[26] Rezaei K., Agahi H., Mahmoodzadeh A. (2020). A Weighted Voting Classifiers Ensemble for the Brain Tumors Classification in MR Images. IETE J. Res..

[27] Fouad A., Moftah H.M., Hefny H.A. (2020). Brain diagnoses detection using whale optimization algorithm based on ensemble learning classifier. Int. J. Intell. Eng. Syst..

[28] Ayadi W., Charfi I., Elhamzi W., Atri M. (2020). Brain tumor classification based on hybrid approach. Vis. Comput..

[29] Srujan K.S., Shivakumar S., Sitnur K., Garde O., Pk P. (2020). Brain Tumor Segmentation and Classification using CNN model. Int. Res. J. Eng. Technol..

[30] Tejaswini G.P., Sreelakshmi K. (2020). Brain Tumour Detection using Deep Neural Network. Wutan Huatan Jisuan Jishu.

[31] Huang Z., Du X., Chen L., Li Y., Liu M., Chou Y., Jin L. (2020). Convolutional Neural Network Based on Complex Networks for Brain Tumor Image Classification with a Modified Activation Function. IEEE Access.

[32] Ghassemi N., Shoeibi A., Rouhani M. (2020). Deep neural network with generative adversarial networks pre-training for brain tumor classification based on MR images. Biomed. Signal Process. Control.

[33] Deepak S., Ameer P.M. (2020). Automated Categorization of Brain Tumor from MRI Using CNN features and SVM. J. Ambient Intell. Humaniz. Comput..

[34] Noreen N., Palaniappan S., Qayyum A., Ahmad I., Alassafi M.O. (2021). Brain Tumor Classification Based on Fine-Tuned Models and the Ensemble Method. Comput. Mater. Contin..

[35] Shaik N.S., Cherukuri T.K. (2022). Multi-level attention network: Application to brain tumor classification. Signal Image Video Process..

[36] Ahmad B., Sun J., You Q., Palade V., Mao Z. (2022). Brain Tumor Classification Using a Combination of Variational Autoencoders and Generative Adversarial Networks. Biomedicines.

[37] Alanazi M.F., Ali M.U., Hussain S.J., Zafar A., Mohatram M., Irfan M., Alruwaili R., Alruwaili M., Ali N.H., Albarrak A.M. (2022). Brain Tumor/Mass Classification Framework Using Magnetic-Resonance-Imaging-Based Isolated and Developed Transfer Deep-Learning Model. Sensors.

[38] Almalki Y.E., Ali M.U., Ahmed W., Kallu K.D., Zafar A., Alduraibi S.K., Irfan M., Basha M.A.A., Alshamrani H.A., Alduraibi A.K. (2022). Robust Gaussian and Nonlinear Hybrid Invariant Clustered Features Aided Approach for Speeded Brain Tumor Diagnosis. Life.

[39] Kavin Kumar K., Dinesh P.M., Rayavel P., Vijayaraja L., Dhanasekar R., Kesavan R., Raju K., Khan A.A., Wechtaisong C., Haq M.A. (2023). Brain Tumor Identification Using Data Augmentation and Transfer Learning Approach. Comput. Syst. Sci. Eng..

[40] Swati Z.N.K., Zhao Q., Kabir M., Ali F., Ali Z., Ahmed S., Lu J. (2019). Brain tumor classification for MR images using transfer learning and fine-tuning. Comput. Med. Imaging Graph..

[41] Ekong F., Yu Y., Patamia R.A., Feng X., Tang Q., Mazumder P., Cai J. (2022). Bayesian Depth-Wise Convolutional Neural Network Design for Brain Tumor MRI Classification. Diagnostics.

[42] Asiri A.A., Shaf A., Ali T., Aamir M., Usman A., Irfan M., Alshamrani H.A., Mehdar K.M., Alshehri O.M., Alqhtani S.M. (2023). Multi-Level Deep Generative Adversarial Networks for Brain Tumor Classification on Magnetic Resonance Images. Intell. Autom. Soft Comput..

[43] Shilaskar S., Mahajan T., Bhatlawande S., Chaudhari S., Mahajan R., Junnare K. Machine Learning Based Brain Tumor Detection and Classification using HOG Feature Descriptor. Proceedings of the International Conference on Sustainable Computing and Smart Systems, ICSCSS.

[44] Yadav S. Analysis of k-fold cross-validation over hold-out validation on colossal datasets for quality classification. Proceedings of the 2016 IEEE 6th International Conference on Advanced Computing (IACC).

[45] Nickparvar M., Brain_Tumor_MRI Dataset (2021). Kaggle. Dataset.

[46] Cheng J., Brain Tumor Dataset (2017). Figshare. https://figshare.com/articles/dataset/brain_tumor_dataset/1512427.

[47] Kaggle Brain Tumor Classification (MRI). https://www.kaggle.com/datasets/sartajbhuvaji/brain-tumor-classification-mri.

[48] Hamada A. (2020). Br35H: Brain Tumor Detection. https://www.kaggle.com/datasets/ahmedhamada0/brain-tumor-detection.

[49] Wang W., Chen Z., Yuan X. (2022). Simple low-light image enhancement based on Weber–Fechner law in logarithmic space. Signal Process. Image Commun..

[50] Wang Y., Su Y., Li W., Xiao J., Li X., Liu A.A. (2023). Dual-path Rare Content Enhancement Network for Image and Text Matching. IEEE Trans. Circuits Syst. Video Technol..

[51] Goodfellow I., Bengio Y., Courville A. (2016). Deep Learning.

[52] Ioffe S., Szegedy C. Batch normalization: Accelerating deep network training by reducing internal covariate shift. Proceedings of the 32nd International Conference on Machine Learning.

[53] Alzubaidi L., Zhang J., Humaidi A.J., Al-Dujaili A., Duan Y., Al-Shamma O., Santamaría J., Fadhel M.A., Al-Amidie M., Farhan L. (2021). Review of Deep Learning: Concepts, CNN Architectures, Challenges, Applications, Future Directions.

[54] Bin Tufail A., Ullah I., Rehman A.U., Khan R.A., Khan M.A., Ma Y.K., Hussain Khokhar N., Sadiq M.T., Khan R., Shafiq M. (2022). On Disharmony in Batch Normalization and Dropout Methods for Early Categorization of Alzheimer’s Disease. Sustainability.

[55] Kingma D.P., Ba J. (2014). Adam: A Method for Stochastic Optimization. Proceedings of the 3rd International Conference on Learning Representations.

[56] Robbins H., Monro S. (1951). A Stochastic Approximation Method. Ann. Math. Stat..

[57] Rasheed Z., Ma Y.-K., Ullah I., Al Shloul T., Bin Tufail A., Ghadi Y.Y., Khan M.Z., Mohamed H.G. (2023). Automated Classification of Brain Tumors from Magnetic Resonance Imaging Using Deep Learning. Brain Sci..

[58] Nair V., Hinton G.E. (2010). Rectified linear units improve Restricted Boltzmann machines. Proceedings of the 27th International Conference on Machine Learning.

[59] Srivastava N., Hinton G., Krizhevsky A., Sutskever I., Salakhutdinov R. (2014). Dropout: A Simple Way to Prevent Neural Networks from Overfitting. J. Mach. Learn. Res..

[60] Moradi R., Berangi R., Minaei B. (2020). A Survey of Regularization Strategies for Deep Models. Artif. Intell. Rev..

[61] ReduceLROnPlateau. https://keras.io/api/callbacks/reduce_lr_on_plateau/.

[62] Simonyan K., Zisserman A. Very deep convolutional networks for large-scale image recognition. Proceedings of the 3rd International Conference on Learning Representations.

[63] Glorot X., Bengio Y. (2010). Understanding the difficulty of training deep feedforward neural networks. J. Mach. Learn. Res..

[64] He K., Zhang X., Ren S., Sun J. Deep residual learning for image recognition. Proceedings of the IEEE Conference on Computer Vision and Pattern Recognition (CVPR).

[65] Sandler M., Howard A., Zhu M., Zhmoginov A., Chen L.C. MobileNetV2: Inverted Residuals and Linear Bottlenecks. Proceedings of the 2018 IEEE Conference on Computer Vision and Pattern Recognition (CVPR).

[66] Szegedy C., Vanhoucke V., Ioffe S., Shlens J., Wojna Z. Rethinking the Inception Architecture for Computer Vision. Proceedings of the 2016 IEEE Computer Society Conference on Computer Vision and Pattern Recognition.

[67] Kuraparthi S., Reddy M.K., Sujatha C.N., Valiveti H., Duggineni C., Kollati M., Kora P., Sravan V. (2021). Brain tumor classification of MRI images using deep convolutional neural network. Trait. Signal.

[68] Ting K.M. (2017). Confusion Matrix. Encyclopedia of Machine Learning and Data Mining.

[69] Hajian-Tilaki K. (2013). Receiver operating characteristic (ROC) curve analysis for medical diagnostic test evaluation. Casp. J. Intern. Med..

